# Supplementary material for: Resolving the full spectrum of human genome variation using Linked-Reads
Source: Genome Res. 2019 Apr;29(4):635–45. doi: 10.1101/gr.234443.118 (PMC6442396; doi:10.1101/gr.234443.118)
Supplement: Supplemental Material [file supp_gr.234443.118_Supplemental_longranger-2.2.zip › longranger-2.2.tar.bz2!/lr2.2-public/lib/stan/cmdstan/stan/doc/api/html/classstan_1_1io_1_1writer.html]

Stan: stan::io::writer< T > Class Template Reference


|  |  |
| --- | --- |
|  | Stan  2.5.0  probability, sampling & optimization |


- Main Page
- Related Pages
- Namespaces
- Classes
- Files

- Class List
- Class Index
- Class Hierarchy
- Class Members

All Classes Namespaces Files Functions Variables Typedefs Enumerations Enumerator Friends Macros Pages

- stan
- io
- writer

Public Types |
Public Member Functions |
Public Attributes |
List of all members

stan::io::writer< T > Class Template Reference

A stream-based writer for integer, scalar, vector, matrix and array data types, which transforms from constrained to a sequence of constrained variables.
More...

`#include <writer.hpp>`

|  |  |
| --- | --- |
| Public Types | |
| typedef Eigen::Matrix< T,   Eigen::Dynamic, Eigen::Dynamic > | matrix\_t |
|  | |
| typedef Eigen::Matrix< T,   Eigen::Dynamic, 1 > | vector\_t |
|  | |
| typedef Eigen::Matrix< T,   1, Eigen::Dynamic > | row\_vector\_t |
|  | |
| typedef Eigen::Array< T,   Eigen::Dynamic, 1 > | array\_vec\_t |
|  | |

|  |  |
| --- | --- |
| Public Member Functions | |
|  | writer (std::vector< T > &data\_r, std::vector< int > &data\_i) |
|  | Construct a writer that writes to the specified scalar and integer vectors. More... |
|  | |
|  | ~writer () |
|  | Destroy this writer. More... |
|  | |
| std::vector< T > & | data\_r () |
|  | Return a reference to the underlying vector of real values that have been written. More... |
|  | |
| std::vector< int > & | data\_i () |
|  | Return a reference to the underlying vector of integer values that have been written. More... |
|  | |
| void | integer (int n) |
|  | Write the specified integer to the sequence of integer values. More... |
|  | |
| void | scalar\_unconstrain (T &y) |
|  | Write the unconstrained value corresponding to the specified scalar. More... |
|  | |
| void | scalar\_pos\_unconstrain (T &y) |
|  | Write the unconstrained value corresponding to the specified positive-constrained scalar. More... |
|  | |
| void | scalar\_lb\_unconstrain (double lb, T &y) |
|  | Return the unconstrained version of the specified input, which is constrained to be above the specified lower bound. More... |
|  | |
| void | scalar\_ub\_unconstrain (double ub, T &y) |
|  | Write the unconstrained value corresponding to the specified lower-bounded value. More... |
|  | |
| void | scalar\_lub\_unconstrain (double lb, double ub, T &y) |
|  | Write the unconstrained value corresponding to the specified value with the specified bounds. More... |
|  | |
| void | corr\_unconstrain (T &y) |
|  | Write the unconstrained value corresponding to the specified correlation-constrained variable. More... |
|  | |
| void | prob\_unconstrain (T &y) |
|  | Write the unconstrained value corresponding to the specified probability value. More... |
|  | |
| void | ordered\_unconstrain (vector\_t &y) |
|  | Write the unconstrained vector that corresponds to the specified ascendingly ordered vector. More... |
|  | |
| void | positive\_ordered\_unconstrain (vector\_t &y) |
|  | Write the unconstrained vector that corresponds to the specified postiive ascendingly ordered vector. More... |
|  | |
| void | vector\_unconstrain (const vector\_t &y) |
|  | Write the specified unconstrained vector. More... |
|  | |
| void | row\_vector\_unconstrain (const vector\_t &y) |
|  | Write the specified unconstrained vector. More... |
|  | |
| void | matrix\_unconstrain (const matrix\_t &y) |
|  | Write the specified unconstrained matrix. More... |
|  | |
| void | vector\_lb\_unconstrain (double lb, vector\_t &y) |
|  | |
| void | row\_vector\_lb\_unconstrain (double lb, row\_vector\_t &y) |
|  | |
| void | matrix\_lb\_unconstrain (double lb, matrix\_t &y) |
|  | |
| void | vector\_ub\_unconstrain (double ub, vector\_t &y) |
|  | |
| void | row\_vector\_ub\_unconstrain (double ub, row\_vector\_t &y) |
|  | |
| void | matrix\_ub\_unconstrain (double ub, matrix\_t &y) |
|  | |
| void | vector\_lub\_unconstrain (double lb, double ub, vector\_t &y) |
|  | |
| void | row\_vector\_lub\_unconstrain (double lb, double ub, row\_vector\_t &y) |
|  | |
| void | matrix\_lub\_unconstrain (double lb, double ub, matrix\_t &y) |
|  | |
| void | unit\_vector\_unconstrain (vector\_t &y) |
|  | Write the unconstrained vector corresponding to the specified unit\_vector value. More... |
|  | |
| void | simplex\_unconstrain (vector\_t &y) |
|  | Write the unconstrained vector corresponding to the specified simplex value. More... |
|  | |
| void | cholesky\_factor\_unconstrain (matrix\_t &y) |
|  | Writes the unconstrained Cholesky factor corresponding to the specified constrained matrix. More... |
|  | |
| void | cholesky\_corr\_unconstrain (matrix\_t &y) |
|  | Writes the unconstrained Cholesky factor for a correlation matrix corresponding to the specified constrained matrix. More... |
|  | |
| void | cov\_matrix\_unconstrain (matrix\_t &y) |
|  | Writes the unconstrained covariance matrix corresponding to the specified constrained correlation matrix. More... |
|  | |
| void | corr\_matrix\_unconstrain (matrix\_t &y) |
|  | Writes the unconstrained correlation matrix corresponding to the specified constrained correlation matrix. More... |
|  | |

|  |  |
| --- | --- |
| Public Attributes | |
| const double | CONSTRAINT\_TOLERANCE |
|  | This is the tolerance for checking arithmetic bounds in rank and in simplexes. More... |
|  | |

## Detailed Description

### template<typename T> class stan::io::writer< T >

A stream-based writer for integer, scalar, vector, matrix and array data types, which transforms from constrained to a sequence of constrained variables.

This class converts constrained values to unconstrained values with mappings that invert those defined in `stan::io::reader` to convert unconstrained values to constrained values.

Template Parameters
:   |  |  |
    | --- | --- |
    | T | Basic scalar type. |

Definition at line 28 of file writer.hpp.

## Member Typedef Documentation

template<typename T >

typedef Eigen::Array<T,Eigen::Dynamic,1> stan::io::writer< T >::array\_vec\_t

Definition at line 38 of file writer.hpp.

template<typename T >

typedef Eigen::Matrix<T,Eigen::Dynamic,Eigen::Dynamic> stan::io::writer< T >::matrix\_t

Definition at line 34 of file writer.hpp.

template<typename T >

typedef Eigen::Matrix<T,1,Eigen::Dynamic> stan::io::writer< T >::row\_vector\_t

Definition at line 36 of file writer.hpp.

template<typename T >

typedef Eigen::Matrix<T,Eigen::Dynamic,1> stan::io::writer< T >::vector\_t

Definition at line 35 of file writer.hpp.

## Constructor & Destructor Documentation

template<typename T >

|  |  |  |  |
| --- | --- | --- | --- |
| stan::io::writer< T >::writer | ( | std::vector< T > & | *data\_r*, |
|  |  | std::vector< int > & | *data\_i* |
|  | ) |  |  |

inline

Construct a writer that writes to the specified scalar and integer vectors.

Parameters
:   |  |  |
    | --- | --- |
    | data\_r | Scalar values. |
    | data\_i | Integer values. |

Definition at line 53 of file writer.hpp.

template<typename T >

|  |  |  |  |  |
| --- | --- | --- | --- | --- |
| stan::io::writer< T >::~writer | ( |  | ) |  |

inline

Destroy this writer.

Definition at line 65 of file writer.hpp.

## Member Function Documentation

template<typename T >

|  |  |  |  |  |  |
| --- | --- | --- | --- | --- | --- |
| void stan::io::writer< T >::cholesky\_corr\_unconstrain | ( | matrix\_t & | *y* | ) |  |

inline

Writes the unconstrained Cholesky factor for a correlation matrix corresponding to the specified constrained matrix.

The unconstraining operation is the inverse of the constraining operation in `cov_matrix_constrain(Matrix<T,Dynamic,Dynamic)`.

Parameters
:   |  |  |
    | --- | --- |
    | y | Constrained covariance matrix. |

Exceptions
:   |  |  |
    | --- | --- |
    | std::runtime\_error | if y has no elements or if it is not square |

Definition at line 426 of file writer.hpp.

template<typename T >

|  |  |  |  |  |  |
| --- | --- | --- | --- | --- | --- |
| void stan::io::writer< T >::cholesky\_factor\_unconstrain | ( | matrix\_t & | *y* | ) |  |

inline

Writes the unconstrained Cholesky factor corresponding to the specified constrained matrix.

The unconstraining operation is the inverse of the constraining operation in `cov_matrix_constrain(Matrix<T,Dynamic,Dynamic)`.

Parameters
:   |  |  |
    | --- | --- |
    | y | Constrained covariance matrix. |

Exceptions
:   |  |  |
    | --- | --- |
    | std::runtime\_error | if y has no elements or if it is not square |

Definition at line 404 of file writer.hpp.

template<typename T >

|  |  |  |  |  |  |
| --- | --- | --- | --- | --- | --- |
| void stan::io::writer< T >::corr\_matrix\_unconstrain | ( | matrix\_t & | *y* | ) |  |

inline

Writes the unconstrained correlation matrix corresponding to the specified constrained correlation matrix.

The unconstraining operation is the inverse of the constraining operation in `corr_matrix_constrain(Matrix<T,Dynamic,Dynamic)`.

Parameters
:   |  |  |
    | --- | --- |
    | y | Constrained correlation matrix. |

Exceptions
:   |  |  |
    | --- | --- |
    | std::runtime\_error | if the correlation matrix has no elements or is not a square matrix. |
    | std::runtime\_error | if the correlation matrix cannot be factorized by factor\_cov\_matrix() or if the sds returned by factor\_cov\_matrix() on log scale are unconstrained. |

Definition at line 483 of file writer.hpp.

template<typename T >

|  |  |  |  |  |  |
| --- | --- | --- | --- | --- | --- |
| void stan::io::writer< T >::corr\_unconstrain | ( | T & | *y* | ) |  |

inline

Write the unconstrained value corresponding to the specified correlation-constrained variable.

The unconstraining transform is `atanh(y)`, which reverses the transfrom in `corr_constrain()`.

Parameters
:   |  |  |
    | --- | --- |
    | y | Correlation value. |

Exceptions
:   |  |  |
    | --- | --- |
    | std::runtime\_error | if y is not between -1.0 and 1.0 |

Definition at line 180 of file writer.hpp.

template<typename T >

|  |  |  |  |  |  |
| --- | --- | --- | --- | --- | --- |
| void stan::io::writer< T >::cov\_matrix\_unconstrain | ( | matrix\_t & | *y* | ) |  |

inline

Writes the unconstrained covariance matrix corresponding to the specified constrained correlation matrix.

The unconstraining operation is the inverse of the constraining operation in `cov_matrix_constrain(Matrix<T,Dynamic,Dynamic)`.

Parameters
:   |  |  |
    | --- | --- |
    | y | Constrained covariance matrix. |

Exceptions
:   |  |  |
    | --- | --- |
    | std::runtime\_error | if y has no elements or if it is not square |

Definition at line 448 of file writer.hpp.

template<typename T >

|  |  |  |  |  |
| --- | --- | --- | --- | --- |
| std::vector<int>& stan::io::writer< T >::data\_i | ( |  | ) |  |

inline

Return a reference to the underlying vector of integer values that have been written.

Returns
:   Values that have been written.

Definition at line 84 of file writer.hpp.

template<typename T >

|  |  |  |  |  |
| --- | --- | --- | --- | --- |
| std::vector<T>& stan::io::writer< T >::data\_r | ( |  | ) |  |

inline

Return a reference to the underlying vector of real values that have been written.

Returns
:   Values that have been written.

Definition at line 73 of file writer.hpp.

template<typename T >

|  |  |  |  |  |  |
| --- | --- | --- | --- | --- | --- |
| void stan::io::writer< T >::integer | ( | int | *n* | ) |  |

inline

Write the specified integer to the sequence of integer values.

Parameters
:   |  |  |
    | --- | --- |
    | n | Integer to write. |

Definition at line 93 of file writer.hpp.

template<typename T >

|  |  |  |  |
| --- | --- | --- | --- |
| void stan::io::writer< T >::matrix\_lb\_unconstrain | ( | double | *lb*, |
|  |  | matrix\_t & | *y* |
|  | ) |  |  |

inline

Definition at line 299 of file writer.hpp.

template<typename T >

|  |  |  |  |
| --- | --- | --- | --- |
| void stan::io::writer< T >::matrix\_lub\_unconstrain | ( | double | *lb*, |
|  |  | double | *ub*, |
|  |  | matrix\_t & | *y* |
|  | ) |  |  |

inline

Definition at line 334 of file writer.hpp.

template<typename T >

|  |  |  |  |
| --- | --- | --- | --- |
| void stan::io::writer< T >::matrix\_ub\_unconstrain | ( | double | *ub*, |
|  |  | matrix\_t & | *y* |
|  | ) |  |  |

inline

Definition at line 316 of file writer.hpp.

template<typename T >

|  |  |  |  |  |  |
| --- | --- | --- | --- | --- | --- |
| void stan::io::writer< T >::matrix\_unconstrain | ( | const matrix\_t & | *y* | ) |  |

inline

Write the specified unconstrained matrix.

Parameters
:   |  |  |
    | --- | --- |
    | y | Matrix to write. |

Definition at line 282 of file writer.hpp.

template<typename T >

|  |  |  |  |  |  |
| --- | --- | --- | --- | --- | --- |
| void stan::io::writer< T >::ordered\_unconstrain | ( | vector\_t & | *y* | ) |  |

inline

Write the unconstrained vector that corresponds to the specified ascendingly ordered vector.

The unconstraining transform is defined for input vector `y` to produce an output vector `x` of the same size, defined by `x[0] = log(y[0])` and by `x[k] = log(y[k] - y[k-1])` for `k > 0`. This unconstraining transform inverts the constraining transform specified in `ordered_constrain(size_t)`.

Parameters
:   |  |  |
    | --- | --- |
    | y | Ascendingly ordered vector. |

Returns
:   Unconstrained vector corresponding to the specified vector.

Exceptions
:   |  |  |
    | --- | --- |
    | std::runtime\_error | if vector is not in ascending order. |

Definition at line 214 of file writer.hpp.

template<typename T >

|  |  |  |  |  |  |
| --- | --- | --- | --- | --- | --- |
| void stan::io::writer< T >::positive\_ordered\_unconstrain | ( | vector\_t & | *y* | ) |  |

inline

Write the unconstrained vector that corresponds to the specified postiive ascendingly ordered vector.

The unconstraining transform is defined for input vector `y` to produce an output vector `x` of the same size, defined by `x[0] = log(y[0])` and by `x[k] = log(y[k] - y[k-1])` for `k > 0`. This unconstraining transform inverts the constraining transform specified in `positive_ordered_constrain(size_t)`.

Parameters
:   |  |  |
    | --- | --- |
    | y | Positive ascendingly ordered vector. |

Returns
:   Unconstrained vector corresponding to the specified vector.

Exceptions
:   |  |  |
    | --- | --- |
    | std::runtime\_error | if vector is not in ascending order. |

Definition at line 241 of file writer.hpp.

template<typename T >

|  |  |  |  |  |  |
| --- | --- | --- | --- | --- | --- |
| void stan::io::writer< T >::prob\_unconstrain | ( | T & | *y* | ) |  |

inline

Write the unconstrained value corresponding to the specified probability value.

The unconstraining transform is `logit(y)`, which inverts the constraining transform defined in `prob_constrain()`.

Parameters
:   |  |  |
    | --- | --- |
    | y | Probability value. |

Exceptions
:   |  |  |
    | --- | --- |
    | std::runtime\_error | if y is not between 0.0 and 1.0 |

Definition at line 195 of file writer.hpp.

template<typename T >

|  |  |  |  |
| --- | --- | --- | --- |
| void stan::io::writer< T >::row\_vector\_lb\_unconstrain | ( | double | *lb*, |
|  |  | row\_vector\_t & | *y* |
|  | ) |  |  |

inline

Definition at line 294 of file writer.hpp.

template<typename T >

|  |  |  |  |
| --- | --- | --- | --- |
| void stan::io::writer< T >::row\_vector\_lub\_unconstrain | ( | double | *lb*, |
|  |  | double | *ub*, |
|  |  | row\_vector\_t & | *y* |
|  | ) |  |  |

inline

Definition at line 329 of file writer.hpp.

template<typename T >

|  |  |  |  |
| --- | --- | --- | --- |
| void stan::io::writer< T >::row\_vector\_ub\_unconstrain | ( | double | *ub*, |
|  |  | row\_vector\_t & | *y* |
|  | ) |  |  |

inline

Definition at line 311 of file writer.hpp.

template<typename T >

|  |  |  |  |  |  |
| --- | --- | --- | --- | --- | --- |
| void stan::io::writer< T >::row\_vector\_unconstrain | ( | const vector\_t & | *y* | ) |  |

inline

Write the specified unconstrained vector.

Parameters
:   |  |  |
    | --- | --- |
    | y | Vector to write. |

Definition at line 271 of file writer.hpp.

template<typename T >

|  |  |  |  |
| --- | --- | --- | --- |
| void stan::io::writer< T >::scalar\_lb\_unconstrain | ( | double | *lb*, |
|  |  | T & | *y* |
|  | ) |  |  |

inline

Return the unconstrained version of the specified input, which is constrained to be above the specified lower bound.

The unconstraining transform is `log(y - lb)`, which inverts the constraining transform defined in `reader::scalar_lb_constrain(double)`,

Parameters
:   |  |  |
    | --- | --- |
    | lb | Lower bound. |
    | y | Lower-bounded value. |

Exceptions
:   |  |  |
    | --- | --- |
    | std::runtime\_error | if y is lower than the lower bound provided. |

Definition at line 136 of file writer.hpp.

template<typename T >

|  |  |  |  |
| --- | --- | --- | --- |
| void stan::io::writer< T >::scalar\_lub\_unconstrain | ( | double | *lb*, |
|  |  | double | *ub*, |
|  |  | T & | *y* |
|  | ) |  |  |

inline

Write the unconstrained value corresponding to the specified value with the specified bounds.

The unconstraining transform is given by `reader::logit((y-L)/(U-L))`, which inverts the constraining transform defined in `scalar_lub_constrain(double,double)`.

Parameters
:   |  |  |
    | --- | --- |
    | lb | Lower bound. |
    | ub | Upper bound. |
    | y | Bounded value. |

Exceptions
:   |  |  |
    | --- | --- |
    | std::runtime\_error | if y is not between the lower and upper bounds |

Definition at line 166 of file writer.hpp.

template<typename T >

|  |  |  |  |  |  |
| --- | --- | --- | --- | --- | --- |
| void stan::io::writer< T >::scalar\_pos\_unconstrain | ( | T & | *y* | ) |  |

inline

Write the unconstrained value corresponding to the specified positive-constrained scalar.

The transformation applied is `log(y)`, which is the inverse of constraining transform specified in `reader::scalar_pos_constrain()`.

This method will fail if the argument is not non-negative.

Parameters
:   |  |  |
    | --- | --- |
    | y | The positive value. |

Exceptions
:   |  |  |
    | --- | --- |
    | std::runtime\_error | if y is negative. |

Definition at line 119 of file writer.hpp.

template<typename T >

|  |  |  |  |
| --- | --- | --- | --- |
| void stan::io::writer< T >::scalar\_ub\_unconstrain | ( | double | *ub*, |
|  |  | T & | *y* |
|  | ) |  |  |

inline

Write the unconstrained value corresponding to the specified lower-bounded value.

The unconstraining transform is `log(ub - y)`, which reverses the constraining transform defined in `reader::scalar_ub_constrain(double)`.

Parameters
:   |  |  |
    | --- | --- |
    | ub | Upper bound. |
    | y | Constrained value. |

Exceptions
:   |  |  |
    | --- | --- |
    | std::runtime\_error | if y is higher than the upper bound provided. |

Definition at line 150 of file writer.hpp.

template<typename T >

|  |  |  |  |  |  |
| --- | --- | --- | --- | --- | --- |
| void stan::io::writer< T >::scalar\_unconstrain | ( | T & | *y* | ) |  |

inline

Write the unconstrained value corresponding to the specified scalar.

Here, the unconstrain operation is a no-op, which matches `reader::scalar_constrain()`.

Parameters
:   |  |  |
    | --- | --- |
    | y | The value. |

Definition at line 104 of file writer.hpp.

template<typename T >

|  |  |  |  |  |  |
| --- | --- | --- | --- | --- | --- |
| void stan::io::writer< T >::simplex\_unconstrain | ( | vector\_t & | *y* | ) |  |

inline

Write the unconstrained vector corresponding to the specified simplex value.

If the specified constrained simplex is of size `K`, the returned unconstrained vector is of size `K-1`.

The transform takes `y = y[1],...,y[K]` and produces the unconstrained vector. This inverts the constraining transform of `simplex_constrain(size_t)`.

Parameters
:   |  |  |
    | --- | --- |
    | y | Simplex constrained value. |

Returns
:   Unconstrained value.

Exceptions
:   |  |  |
    | --- | --- |
    | std::runtime\_error | if the vector is not a simplex. |

Definition at line 382 of file writer.hpp.

template<typename T >

|  |  |  |  |  |  |
| --- | --- | --- | --- | --- | --- |
| void stan::io::writer< T >::unit\_vector\_unconstrain | ( | vector\_t & | *y* | ) |  |

inline

Write the unconstrained vector corresponding to the specified unit\_vector value.

If the specified constrained unit\_vector is of size `K`, the returned unconstrained vector is of size `K-1`.

The transform takes `y = y[1],...,y[K]` and produces the unconstrained vector. This inverts the constraining transform of `unit_vector_constrain(size_t)`.

Parameters
:   |  |  |
    | --- | --- |
    | y | Simplex constrained value. |

Returns
:   Unconstrained value.

Exceptions
:   |  |  |
    | --- | --- |
    | std::runtime\_error | if the vector is not a unit\_vector. |

Definition at line 358 of file writer.hpp.

template<typename T >

|  |  |  |  |
| --- | --- | --- | --- |
| void stan::io::writer< T >::vector\_lb\_unconstrain | ( | double | *lb*, |
|  |  | vector\_t & | *y* |
|  | ) |  |  |

inline

Definition at line 289 of file writer.hpp.

template<typename T >

|  |  |  |  |
| --- | --- | --- | --- |
| void stan::io::writer< T >::vector\_lub\_unconstrain | ( | double | *lb*, |
|  |  | double | *ub*, |
|  |  | vector\_t & | *y* |
|  | ) |  |  |

inline

Definition at line 324 of file writer.hpp.

template<typename T >

|  |  |  |  |
| --- | --- | --- | --- |
| void stan::io::writer< T >::vector\_ub\_unconstrain | ( | double | *ub*, |
|  |  | vector\_t & | *y* |
|  | ) |  |  |

inline

Definition at line 306 of file writer.hpp.

template<typename T >

|  |  |  |  |  |  |
| --- | --- | --- | --- | --- | --- |
| void stan::io::writer< T >::vector\_unconstrain | ( | const vector\_t & | *y* | ) |  |

inline

Write the specified unconstrained vector.

Parameters
:   |  |  |
    | --- | --- |
    | y | Vector to write. |

Definition at line 260 of file writer.hpp.

## Member Data Documentation

template<typename T >

const double stan::io::writer< T >::CONSTRAINT\_TOLERANCE

This is the tolerance for checking arithmetic bounds in rank and in simplexes.

The current value is `1E-8`.

Definition at line 44 of file writer.hpp.

---

The documentation for this class was generated from the following file:

- src/stan/io/writer.hpp


---

[ Stan Home Page ]

*© 2011–2014,
Stan Development Team.*
